# Jia-Wei-Si-Miao-Yong-An decoction modulates intestinal flora and metabolites in acute coronary syndrome model

**DOI:** 10.3389/fcvm.2022.1038273

**Published:** 2023-01-04

**Authors:** Ning Zhao, Ying Wang, Yan Ma, Xiaoxue Liang, Xi Zhang, Yuan Gao, Yingying Dong, Dong Bai, Jingqing Hu

**Affiliations:** ^1^Formula-Syndrome Research Center, Institute of Basic Theory for Chinese Medicine, China Academy of Chinese Medical Sciences, Beijing, China; ^2^Department of Pharmacy, Xiyuan Hospital, China Academy of Chinese Medical Sciences, Beijing, China; ^3^Department of Pathophysiology and Allergy Research, Vienna General Hospital, Center of Pathophysiology, Infectiology and Immunology, Medical University of Vienna, Vienna, Austria

**Keywords:** ACS, TCM, Si-Miao-Yong-An decoction, gut flora, metabolomics

## Abstract

**Aims:**

We assessed the efficacy of the traditional Chinese medicine formulation Jia-Wei-Si-Miao-Yong-An decoction (HJ11) in the treatment of acute coronary syndrome and evaluated its impact on the intestinal microbiota and their metabolites.

**Methods:**

An acute coronary syndrome model was established in rats, which were randomly assigned to the model, HJ11 treatment, and atorvastatin treatment groups. Rats were then administered saline solution (model and sham operation control groups) or drugs by oral gavage for 28 d. Echocardiography was performed and serum creatine kinase-MB and cardiac troponin I levels were monitored to examine the cardiac function. Inflammation was evaluated using hematoxylin and eosin staining of heart tissue, and serum interleukin-2, interleukin-6, tumor necrosis factor alpha, and high-sensitivity C-reactive protein measurements. Gut microbiota composition was analyzed *via* 16S rRNA gene sequencing. Metabolomics was used to determine fecal metabolites and elucidate the modes of action of HJ11 in acute coronary syndrome treatment.

**Results:**

HJ11 improved cardiac function and attenuated inflammation in rats with acute coronary syndrome. Relative to the untreated model group, the HJ11-treated group presented normalized Firmicutes/Bacteroidetes ratio and reduced abundances of the bacterial genera *norank_f__Ruminococcaceae, Desulfovibrio, Clostridium_sensu_stricto_1, Adlercreutzia, Staphylococcus, Bacteroides, Prevotella, Rikenellaceae_RC9_gut_group, unclassified_o__Bacteroidales*, and *Ruminococcus_gauvreauii_group*. We found 23 differentially expressed intestinal metabolites, and the enriched metabolic pathways were mainly related to amino acid metabolism. We also discovered that asymmetric dimethylarginine levels were strongly associated with cardiovascular disease. Correlation analyses revealed strong associations among intestinal microflora, their metabolites, proinflammatory factors, and cardiac function. Hence, the therapeutic effects of HJ11 on acute coronary syndrome are related to specific alterations in gut microbiota and their metabolites.

**Conclusion:**

This work demonstrated that HJ11 effectively treats acute coronary syndrome. HJ11 seems to increase the abundance of beneficial bacterial taxa (*Bacteroides* and *Rikenellaceae_RC9_gut*_group), mitigate the risk factors associated with cardiovascular disease, alter bacterial metabolites, lower asymmetric dimethylarginine levels, and effectively treat acute coronary syndrome.

## 1. Introduction

Acute coronary syndrome (ACS) is a severe form of heart disease. Its incidence and mortality rate are high and annually increasing, imposing a heavy financial burden on health care systems worldwide (1). ACS is mainly characterized by destabilization, fragmentation, and dislodgement of coronary atherosclerotic plaques, thrombosis, and coronary arteries luminal narrowing or occlusion, being a rapidly progressing disease with poor prognosis (2).

The function of coronary vascular endothelial cells is highly affected by endothelial injury and inflammatory factors (3). Moreover, the inflammatory response plays an important role in ACS development (4) and, according to traditional Chinese medicine (TCM) theory, “inflammatory” is closely related to “heat toxicity” in the pathogenesis of coronary heart disease (5). The intestinal microflora has also been shown to affect the vasculature inflammatory state (6). Hence, restoring the intestinal microflora homeostasis might prevent and treat cardiovascular disease (CVD) (7).

TCM has shown certain advantages over Western medicine in the treatment of ACS (8–11). Systematic clinical and experimental studies have been conducted on the renowned TCM therapeutic Si-Miao-Yong-An decoction (SM) and its administration for the treatment of CVDs such as ACS (12, 13). SM consists of *Lonicerae japonicae* flos, *Scrophularia ningpoensis* Hemsl., *Angelicae sinensis* Radix, and *Glycyrrhizae* Radix et Rhizoma. Jia-Wei-Si-Miao-Yong-An decoction (HJ11), which contains *Forsythiae* Fructus, *Salvia miltiorrhiza* Bunge, *Cinnamomum osmophloeum*, and *Polygoni cuspidati* Rhizoma et Radix in addition to the herbs present in SM, is a formulation developed based on the TCM theory and clinical practice. SM was found to downregulate proinflammatory factors, such as oxidized low-density lipoproteins, interleukin (IL)-6, tumor necrosis factor alpha (TNF-α), and C-reactive protein (CRP), promote endothelial repair, and inhibit thrombosis (14, 15). Certain herbs in SM attenuate the inflammatory response, protect endothelial function, regulate lipid metabolism, stabilize plaque, and improve cardiac function (16–18). Furthermore, most herbs in SM modulate the intestinal microflora (19–23). However, the effects of SM and HJ11 on the intestinal microflora and ACS are unknown.

**Figure 1:**
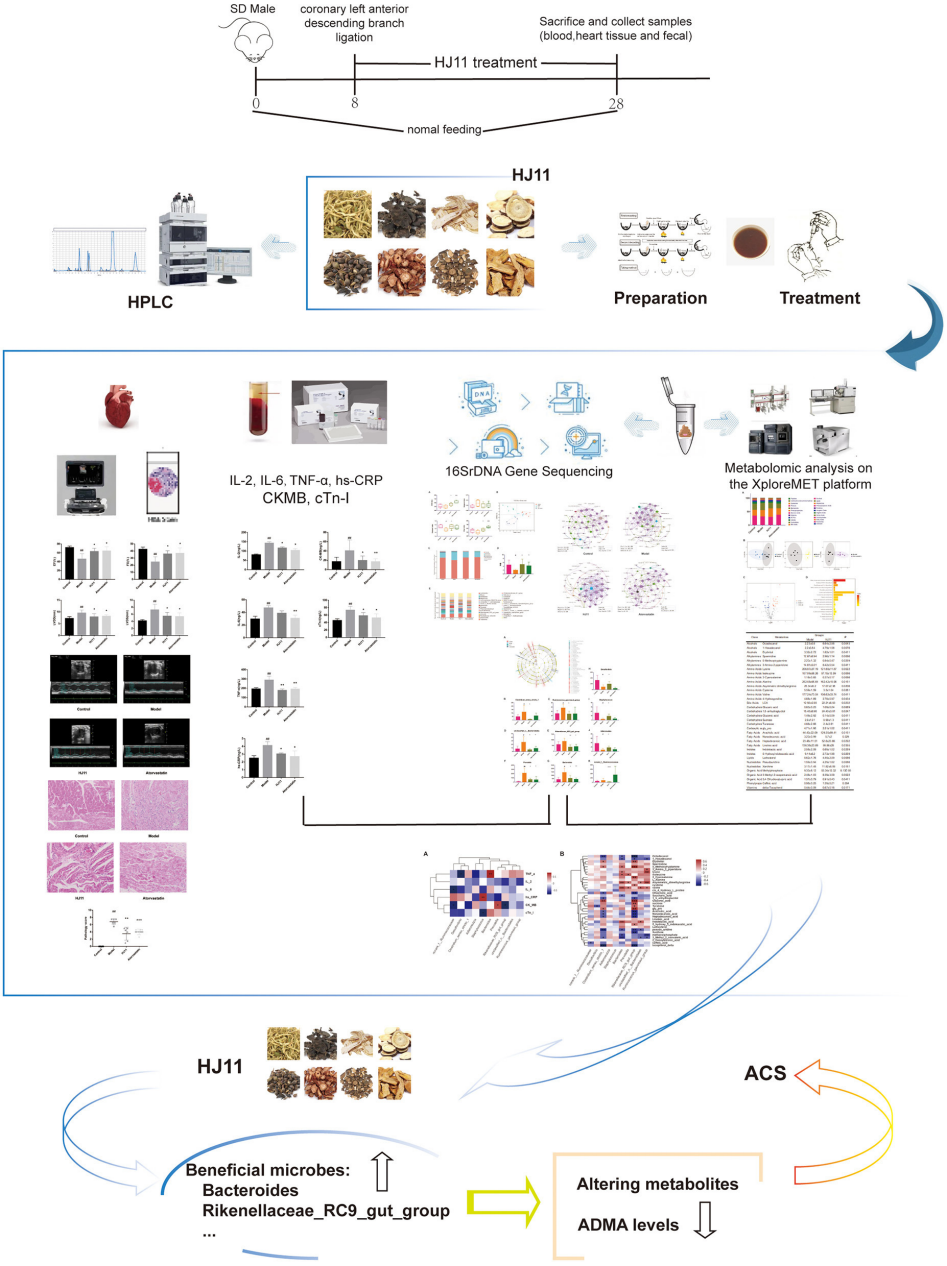
Workflow for predicting the therapeutic mechanism of HJ11 in ACS.

In this study, we investigated the ability of HJ11 to regulate the intestinal microflora and metabolites in rats with induced ACS (Figure 1). Our objective was to lay theoretical and empirical foundations for the clinical application and efficacy improvement of TCM in ACS therapy.

## 2. Materials and methods

### 2.1 Materials

#### 2.1.1 Instruments

The apparatus and equipment used in the experiments included an animal ventilator (HX-200; Chengdu Taimeng Technology Co. Ltd., Chengdu, China), biosignal and pressure measurement system (RM6240BD; Chengdu Instrument Factory, Chengdu, China), small animal ultrasound imaging system (P6-VET; Jiangsu Dawei Medical Co. Ltd., Jiangsu, China), time-of-flight mass spectrometer (Pegasus HT; Leco Corp., St. Joseph, MO, USA), Agilent 7890B gas chromatograph (Gerstel, Muehlheim, Germany), RXI-5 MS capillary column (30 m × 250 μm i.d., 0.25 μm-thick film; Restek Corporation, Bellefonte, PA, USA), Repeater Xstream electronic pipette (Eppendorf, Hamburg, Germany), hemostatic forceps, forceps, needle holders, tissue and eye scissors, rat surgical table with fixed plates, and cold light source.

#### 2.1.2 Reagents and drugs

The herbs used in the experiments were purchased from the Beijing Shuangqiao Yanjing Traditional Chinese Medicine Company (Beijing, China). *L. japonicae* flos was produced in Shandong, China (production batch No. 2010007); *Forsythiae* Fructus was produced in Shanxi, China (production batch No. 20041001); *S. ningpoensis* Hemsl. was produced in Hebei, China (production batch No. 20112103); *C. osmophloeum* was produced in Fujian, China (production batch No. 20131101); *A. sinensis* Radix was produced in Gansu, China (production batch No. 20100018); *S. miltiorrhiza* Bunge was produced in Shandong, China (production batch No. 2010009); *Glycyrrhizae* Radix et Rhizoma was produced in Xinjiang, China (production batch No. 20090201); and *P. cuspidati* Rhizoma et Radix was produced in Hunan, China (production batch No. 20072901). All herbs were authenticated by the China Academy of Chinese Medical Sciences (CACMS). Atorvastatin (production batch No. S56741) was purchased from the Xiyuan Hospital CACMS, Beijing, China.

Methoxyamine hydrochloride, fatty acid methyl ester (C7–C30) standards, pyridine, and anhydrous sodium sulfate were obtained from Sigma-Aldrich (St. Louis, MO, USA). *N*-methyl-*N*- (trimethylsilyl) trifluoroacetamide with 1% (v/v) trimethylchlorosilane, methanol (Optima LC-MS), acetonitrile (Optima LC-MS), hexane, dichloromethane, chloroform, and acetone were purchased from Thermo Fisher Scientific (Fairlawn, NJ, USA). Ultrapure water was generated using a Milli-Q Reference System fitted with the LC-MS Pak filter (EMD Millipore, Billerica, MA, USA).

The following enzyme-linked immunosorbent assay (ELISA) kits were purchased from the Nanjing Jiancheng Bioengineering Institute, Jiangsu, China: creatine kinase-MB (CK-MB; lot No. R20210327), cardiac troponin I (cTnI; lot No. R20210327), high-sensitivity CRP (hs-CRP; lot No. R20210328), IL-2 (lot No. R20210328), IL-6 (lot No. R20210328), and TNF-α (lot No. R20210328). The PowerSoil^®^ DNA Isolation Kit was purchased from VWR (Radnor, PA, USA).

#### 2.1.3 Animals

Male Sprague–Dawley rats (*n* = 48, weight 200 ± 20 g) were purchased from the China Academy of Food and Drug Administration (Beijing,China) under license No. SCXK (Beijing) 2017-0005. They were housed at the Institute of Basic Theory for Chinese Medicine of the CACMS in a specific pathogen-free laboratory at 20–25°C, 40–60% relative humidity, 12 h light/12 h dark cycle, and with *ad libitum* food and water access. The chow was purchased from Beijing Keao Company (Beijing,China) under license No. Beijing Feeding Certificate 2018-06073. All procedures were in accordance with the National Institute of Health Guide for the Use and Care of Laboratory Animals, and were approved by the Animal Ethics Committee of the Institute of Basic Theory for Chinese Medicine (CACMS) (ethics approval No. IBTCMCACMS21-1903-01) [128].

#### 2.1.4 Drug preparation

The HJ11 formula was composed of *L. japonicae* flos (15 g), *S. ningpoensis* Hemsl. (15 g), *A. sinensis* Radix (15 g), *Glycyrrhizae* Radix et Rhizoma (9 g), *Forsythiae* Fructus (15 g), *S. miltiorrhiza* Bunge (15 g), *C. osmophloeum* (15 g), and *P. cuspidati* Rhizoma et Radix (9 g). The herbs were combined with 500 ml distilled water, soaked for 30 min, and decocted at atmospheric pressure for 30 min. Dregs were filtered, the concentration was adjusted to 1 g raw herbs/mL, and the diluted decoction was stored at 4°C.

Atorvastatin was pulverized and dissolved in distilled water. The solution concentration was adjusted to 1 mg/mL, and the dilution was stored at 4°C.

### 2.2 Methods

#### 2.2.1 Determination of major components

The HJ11 components were determined according to the *Chinese Pharmacopeia 2020* directives regarding the content requirements of the main components of TCM herbs and component detection method. High-performance liquid chromatography (HPLC) was used for quality control testing.

#### 2.2.2 Animal grouping and treatment

The ACS rat model was established by performing ligation of the left anterior descending branch of the coronary artery (24). Successfully modeled, surviving rats were randomly assigned to the model, HJ11, and atorvastatin groups (*n* = 6/group). Rats that survived the sham operation were assigned to the control group (*n* = 6). Rats were administered 7 g HJ11/kg/d (HJ11 group) (19), 10 mg atorvastatin/kg/d (atorvastatin group), or saline (0.9% [w/v] NaCl; control and model groups) by oral gavage for 28 consecutive days.

#### 2.2.3 General state observation

Changes in body weight, activity, hair luster, and diet were recorded. Body weight growth rates were calculated as follows:


$$\begin{array}{l} \text{Body weight growth rate}=\frac{\text{finalweight }-\text{ startingweight}}{\text{startingweight}}\\ \;\times 100\% \end{array}$$

#### 2.2.4 Echocardiography

Echocardiography was performed to evaluate cardiac function 28 d after the treatments. The rats were placed supine on a thermostatic heating plate, and their extremities were fixed with adhesive tape. The transducer frequency was 10 MHz. The echocardiograms were recorded at the papillary muscle level in the left ventricular short axis. The parameters measured included heart rate, ejection factor (EF), fractional shortening (FS), and left ventricular internal end-diastolic dimension (LVDD), and left ventricular internal end-systolic dimension (LVDS).

#### 2.2.5 Biochemical parameters

After 28 d of treatment, the rats were fasted without water for 12 h and anesthetized intraperitoneally using 3% (w/v) sodium pentobarbital. Then, 5 mL blood was taken from the abdominal aorta, centrifuged at 1,000 rpm 4°C for 10 min, and the serum was collected. The latter was analyzed using ELISA to quantify CK-MB, cTnI, IL-6, IL-2, hs-CRP, and TNF-α levels.

#### 2.2.6 Pathological observations

Hematoxylin and eosin (HE) staining was used to observe the myocardial histomorphology. At the end of the experiment, the animals were anesthetized intramuscularly using sodium pentobarbital (30 mg/kg) and sacrificed. The left ventricular tissues were excised, fixed in 10% (v/v) neutral formaldehyde for 1 week, dehydrated using ethanol gradient series, embedded in paraffin, sectioned, and stained with HE. The myocardial histomorphology was observed under a microscope and photographed. Cardiac tissue lesion assessment criteria included myocardial fibrosis, myocardial fibrotic necrosis, and interstitial inflammatory cell infiltration. Lesions were scored 0–4, from none to severe. All scores were cumulative, and the mean score was “X ± SD.”

#### 2.2.7 Intestinal flora analysis using 16S rDNA sequencing

Fresh fecal samples were collected from the ilea of sacrificed rats. Fecal DNA was extracted using the PowerSoil^®^ DNA Isolation Kit and verified on 1% (w/v) agarose gel. DNA concentration and purity were determined using a NanoDrop 2000 UV-vis spectrophotometer (Thermo Fisher Scientific, Wilmington, DE, USA). PCR amplification of the 16S rDNA sequences was performed using primer sets specific for the V3–V4 variable regions. The PCR product was extracted using 2% (w/v) agarose gel and purified using an AxyPrep^®^ DNA Gel Extraction Kit (Axygen Biosciences, Union City, CA, USA) according to manufacturer's instructions. The PCR product was then quantified using a Quantus™ fluorometer (Promega, Madison, WI, USA). The purified amplicons were pooled in equimolar quantities and paired-end sequenced on an Illumina MiSeq PE300 platform/NovaSeq PE250 platform (Illumina, San Diego, CA, USA) according to the standard protocols by Majorbio Bio-Pharm Technology Co. Ltd. (Shanghai, China).The raw sequencing reads were deposited into the NCBI Sequence Read Archive (SRA) database.

Fastp 0.19.6^1^, FLASH 1.2.11^2^, UPARSE 7.1^3^, RDP classifier 2.11^4^, and PICRUSt2 2.2.0^5^ were used to cluster and analyze the valid sequences comprising the operational taxonomic unit set. The latter was then subjected to taxonomic analysis. Information regarding the bacterial species richness and evenness of each sample was obtained based on the results of the operational taxonomic unit analysis. Microbial α- and β-diversity analyses, and linear discriminant analysis effect size were then performed for each sample. A sample grouping analysis was performed based on a partial least squares discriminant analysis (PLS-DA). The bacterial community structure was statistically analyzed based on the taxonomic information, and its correlation with CK-MB, cTnI, hs-CRP, IL-2, IL-6, and TNF-α levels, and other clinical factors was determined *via* Spearman's rank correlation. Correlation heatmaps were then plotted. Differences among groups were determined using the Student's *t*-test. R language and Gephi 0.9.2 in a network analysis were used to unveil the differences in gut microbiota complexity among and within groups.

#### 2.2.8 Metabolomics analysis of the intestinal content

Untargeted metabolomics profiling was performed on the XploreMET platform (Metabo-Profile, Shanghai, China). Approximately 100 mg of each fecal sample from the ilea was placed in a centrifuge tube, frozen, stored in an Eppendorf SafeLock microcentrifuge tube (Eppendorf), and mixed with 25 mg pre-chilled zirconium oxide beads and 10 μL internal standard. Each 50 μL aliquot of 50% (v/v) pre-chilled methanol was added for automated homogenization. The suspensions were centrifuged at 14,000 × g and 4°C for 20 min, and the supernatants were transferred to autosampler vials. Each 175 μL aliquot of pre-chilled methanol/chloroform (3:1 [v/v]) was added to the residue for the second extraction. The suspensions were centrifuged at 14,000 × g and 4°C for 20 min, and each 100 μL supernatant was transferred to an autosampler vial. All samples in the autosampler vials were briefly evaporated using a CentriVap vacuum concentrator (Labconco Corp., Kansas City, MO, USA) to remove chloroform and lyophilized using a FreeZone freeze dryer (Labconco) fitted with a stopping tray dryer. Samples were derivatized and injected *via* a robotic MultiPurposeSampler MPS Dual Head. Each dried sample was derivatized using 50 μL methoxyamine (20 mg/mL in pyridine) at 30°C for 2 h, followed by 50 μL *N*-methyl-*N*-(trimethylsilyl)trifluoroacetamide (1% trimethylchlorosilane) at 37.5°C for another 1 h using the sample preparation head. The derivatized samples were then injected in parallel using a sample injection head.

Helium was the carrier gas, and the flow rate was a constant 1.0 mL/min. The temperature of the injection and transfer interface was 270°C, and the source temperature was 220°C. Measurements were made using electron impact ionization (70 eV) in full scan mode (m/z 50–500).

Test mixtures, internal standards, retention indices, and pooled biological quality control samples were routinely used on the comprehensive metabolomics platform. ChromaTOF software was used to annotate the metabolites by comparing the retention indices and mass spectral data against those previously generated from reference standards with known structures and appearing in the JiaLib metabolite database (Metabo-Profile).

#### 2.2.9 Statistical analyses

The experimental data were expressed as mean ± standard deviation. GraphPad Prism 7.0 (GraphPad Software Inc., La Jolla, CA, USA) was used to plot data, while the SPSS v. 21.0 (SPSS Inc., Chicago, IL, USA) statistical software was used to process data. Weight, cardiac ultrasound, and serum indices were compared among multiple treatment groups using one-way analysis of variance. Pairwise comparisons were made using the Student's *t*-test. *P* < 0.05 was considered statistically significant.

## 3. Results

### 3.1 Determination of major components in HJ11

**Figure 2:**
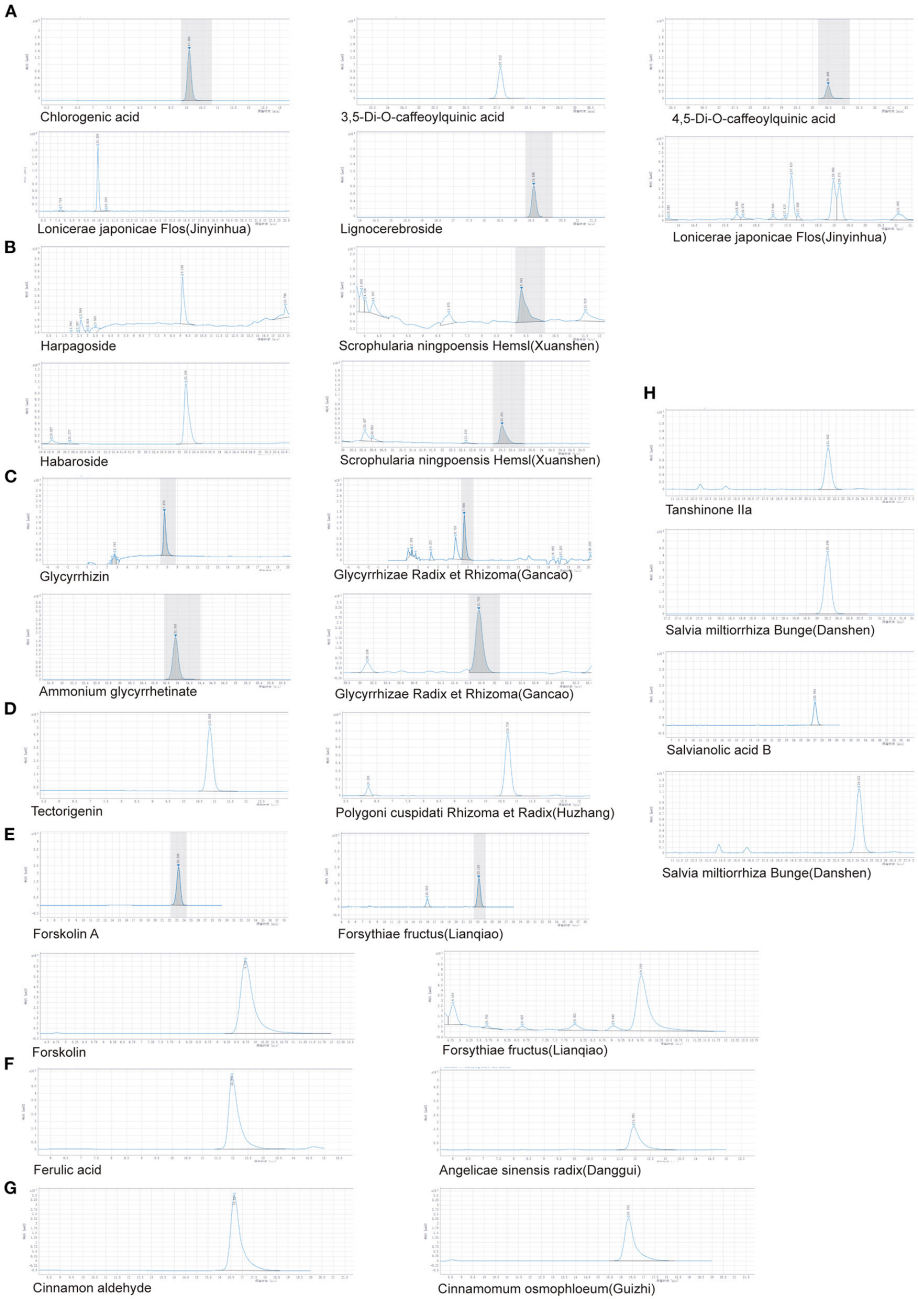
HPLC determination of major components in different herbs. **(A)** *Lonicerae japonicae* flos (Jinyinhua). **(B)** *Scrophularia ningpoensis* Hemsl. (Xuanshen). **(C)** *Glycyrrhizae* Radix et Rhizoma (Gancao). **(D)** *Polygoni cuspidati* Rhizoma et Radix (Huzhang). **(E)** *Forsythiae* Fructus (Lianqiao). **(F)** *Angelicae sinensis* Radix (Danggui). **(G)** *Cinnamomum osmophloeum* (Guizhi). **(H)** *Salvia miltiorrhiza* Bunge (Danshen).

HPLC was used to identify the components in HJ11. Based on the Chinese Pharmacopeia 2020 requirements and detection methods of the main ingredients of Chinese herbs, we analyzed eight herbs and 18 components, and found that all met the required standards. The results are shown in Figure 2 and Table 1.

**Table 1:** Determination of major components in different herbs (*n* = 3).

**Herb**	**Components**	**Measured content (%)**	**Minimum content standard (%)**
*Lonicerae japonicae* flos (Jinyinhua)	Chlorogenic acid	2.82	1.50
	Chlorogenic acid, 3,5-di-O-caffeoylquinic acid, and 4,5-di-O-caffeoylquinic acid	4.79	3.80
	Lignocerebroside	0.07	0.05
*Scrophularia ningpoensis* Hemsl. (Xuanshen)	Harpagoside and habaroside	1.28	0.45
*Angelicae sinensis* Radix (Danggui)	Ferulic acid	0.08	0.05
*Glycyrrhizae* Radix et Rhizoma (Gancao)	Glycyrrhizin	1.07	0.5
	Ammonium glycyrrhetinate	2.54	2.00
*Forsythiae* Fructus (Lianqiao)	Forskolin	0.44	0.15
	Forskolin A	9.02	3.50
*Salvia miltiorrhiza* Bunge (Danshen)	Tanshinone IIa,	0.32	0.25
	Salvianolic acid B	5.92	3.00
*Cinnamomum osmophloeum* (Guizhi)	Cinnamon aldehyde	1.42	1.00
*Polygoni cuspidati* Rhizoma et Radix (Huzhang)	Rhodopsin	4.65	0.60
	Tectorigenin	1.96	0.15

### 3.2 Effects of HJ11 on cardiac function and serum inflammatory cytokine levels

**Figure 3:**
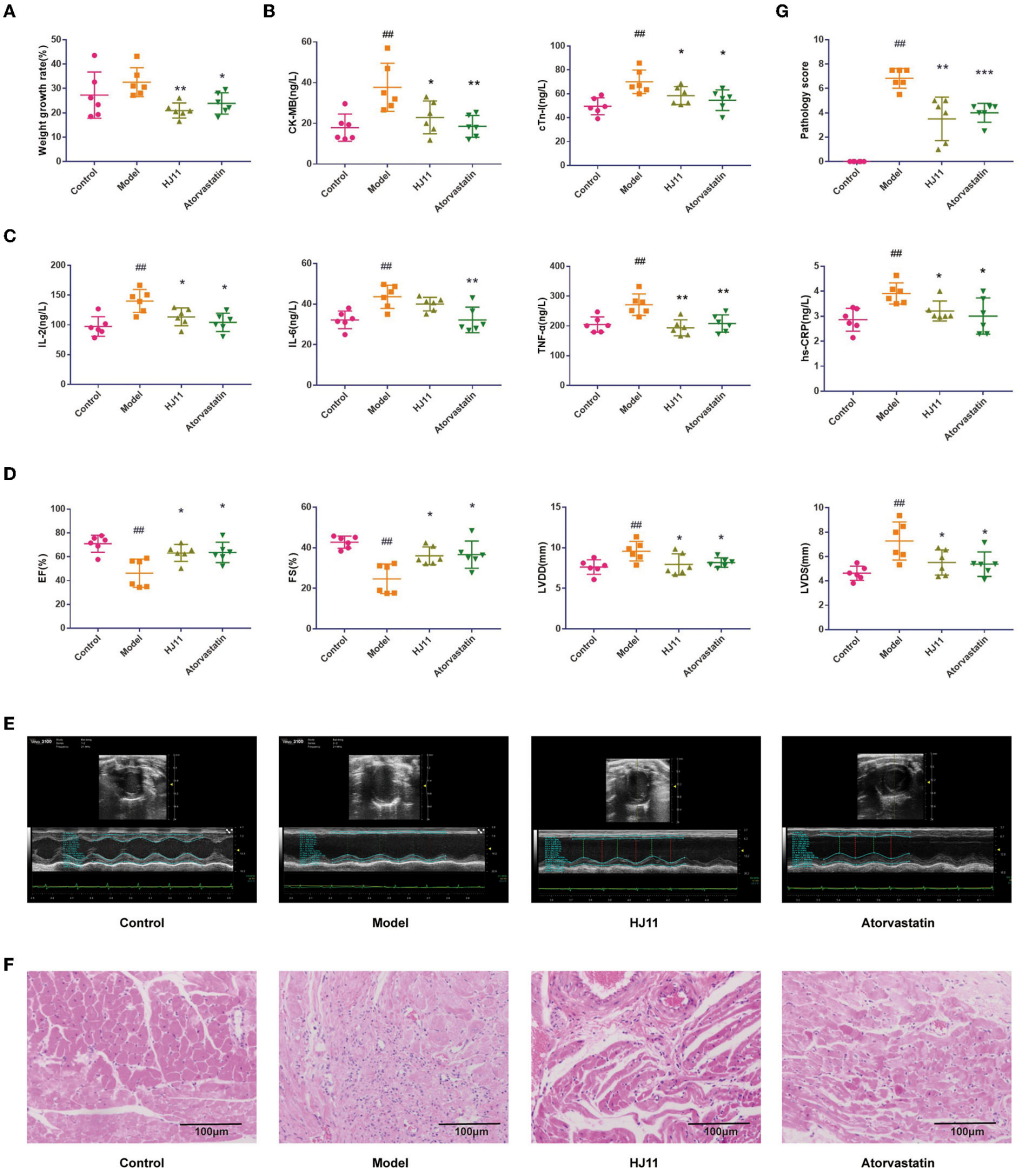
Effects of HJ11 on ACS model rats. **(A)** Changes in body weight in different groups. **(B)** Cardiac enzyme detection in different groups. **(C)** Inflammatory factor detection in different groups. **(D)** Echocardiogram data. **(E)** Echocardiogram images. **(F)** HE staining of heart tissue. **(G)** Pathological scores of heart tissue. ^##^*P* < 0.01 compared with the control group; ^*^P < 0.05, ^**^P < 0.01, ^***^P < 0.001 compared with the model group.

There were no significant differences among groups in terms of activity, hair, or diet. Compared with the model group, the HJ11 (*P* < 0.01) and atorvastatin (*P* < 0.05) groups showed reduced body weight growth rates (Figure 3A). HJ11 reversed the effects of ACS on the serum CK-MB and cTnI levels (Figure 3B). It also significantly reduced the serum IL-2, TNF-α, and hs-CRP levels but not that of serum IL-6 (Figure 3C). The echocardiography results are shown in Figures 3D, E. Compared with the model group, the HJ11 group exhibited increased EF and FS (*P* < 0.05), and decreased left ventricular internal end-diastolic dimension and -systolic dimension (*P* < 0.05). These results suggest that HJ11 lowers proinflammatory cytokine levels and ameliorates the ACS-induced damage to cardiac function.

### 3.3 Histopathological examination

The histopathological examination and score (Figures 3F,G) showed that the model group had significant cardiac lesions compared to the control group (*P* < 0.01), including disorganized myocardial tissue, broken myocardial bundles, widened gaps, myocardial cell degeneration and damage, massive inflammatory cell infiltration in the interstitium, nuclear consolidation and deviation, and blood vessel proliferation, dilatation, and hemorrhage. In contrast, the cardiac lesions in the HJ11 group showed amelioration of myocardial fiber degeneration/necrosis, myocardial fibrosis, and interstitial inflammatory cell infiltration compared to those in the model group (*P* < 0.01).

### 3.4 HJ11 regulation of intestinal flora in rats with ACS

**Figure 4:**
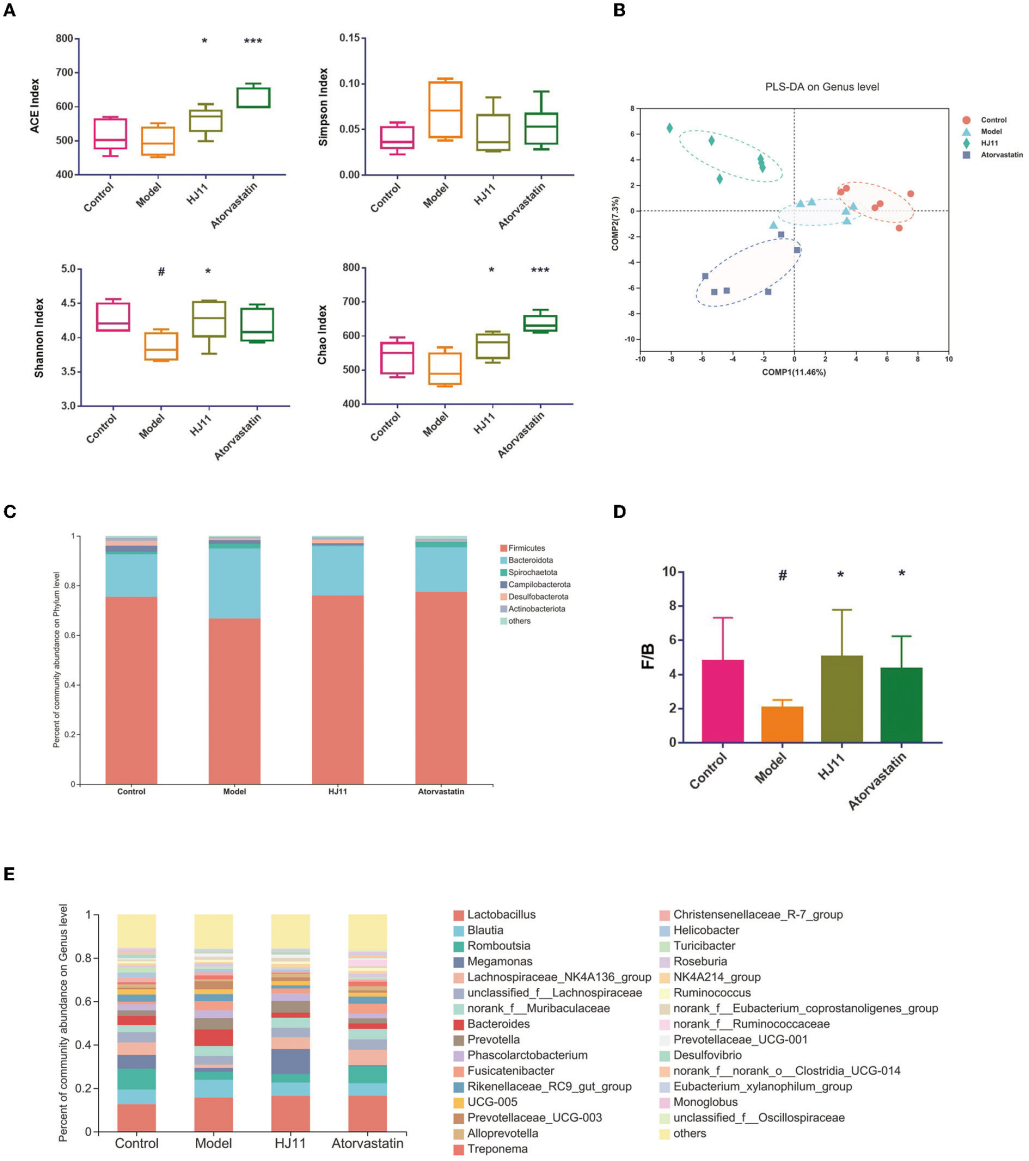
Microbial community structures in different groups. **(A)** α-Diversity in different groups. **(B)** PLS-DA of different groups. Points with the same color and shape represent samples from the same group. Distances between points reflect differences between samples. **(C)** Comparison of community composition at the phylum level. Each column represents a group. **(D)** F/B ratio. **(E)** Comparison of community composition at the genus level. Each column represents a group. ^#^*P* < 0.05, ^#^*P* < 0.01 compared with the control group; *P < 0.05, ***P < 0.001 compared with the model group.

We sequenced the 16S rRNA gene to evaluate the effects of HJ11 on the intestinal flora in ACS rats. Systematic bioinformatics analysis showed that HJ11 stabilized the gut microbial community structure and composition. The α-diversity was estimated to investigate the relative changes in gut microbiota (GM). The Ace and Chao indices are operational taxonomic unit values representing GM species richness, while the Shannon and Simpson indices are quantitative indices. Compared with the control group, the model group presented a significantly reduced Shannon index, whereas the remaining indices did not significantly change between these two groups. Compared with the model group, the HJ11 group displayed restored Ace, Chao, and Shannon indices (Figure 4A), suggesting that HJ11 improves gut microbial diversity in ACS rats.

PLS-DA was used to assess sample grouping (Figure 4B). The four groups were separated, with no crossover. An analysis of similarities was conducted to judge whether sample grouping was meaningful and revealed significant differences between groups (*P* = 0.001).

Differential microbiota analyses were performed on various bacterial taxa. At the phylum level, the groups with the highest relative abundances were Firmicutes, Bacteroidetes, Spirochaetota, Campylobacterota, Desulfobacterota, and Actinobacterota, with Firmicutes and Bacteroidetes being the predominant ones (90%) (Figure 4C). The Firmicutes/Bacteroidetes (F/B) ratio in the HJ11 group differed from that in the control group (Figure 4D). Moreover, there were substantial differences among groups in terms of bacterial genera (Figure 4E), we speculate that these changed bacterial genera may be related to HJ11 treatment of ACS model rats, which merits further investigation.

### 3.5 Specific microbiota in different treatment groups

**Figure 5:**
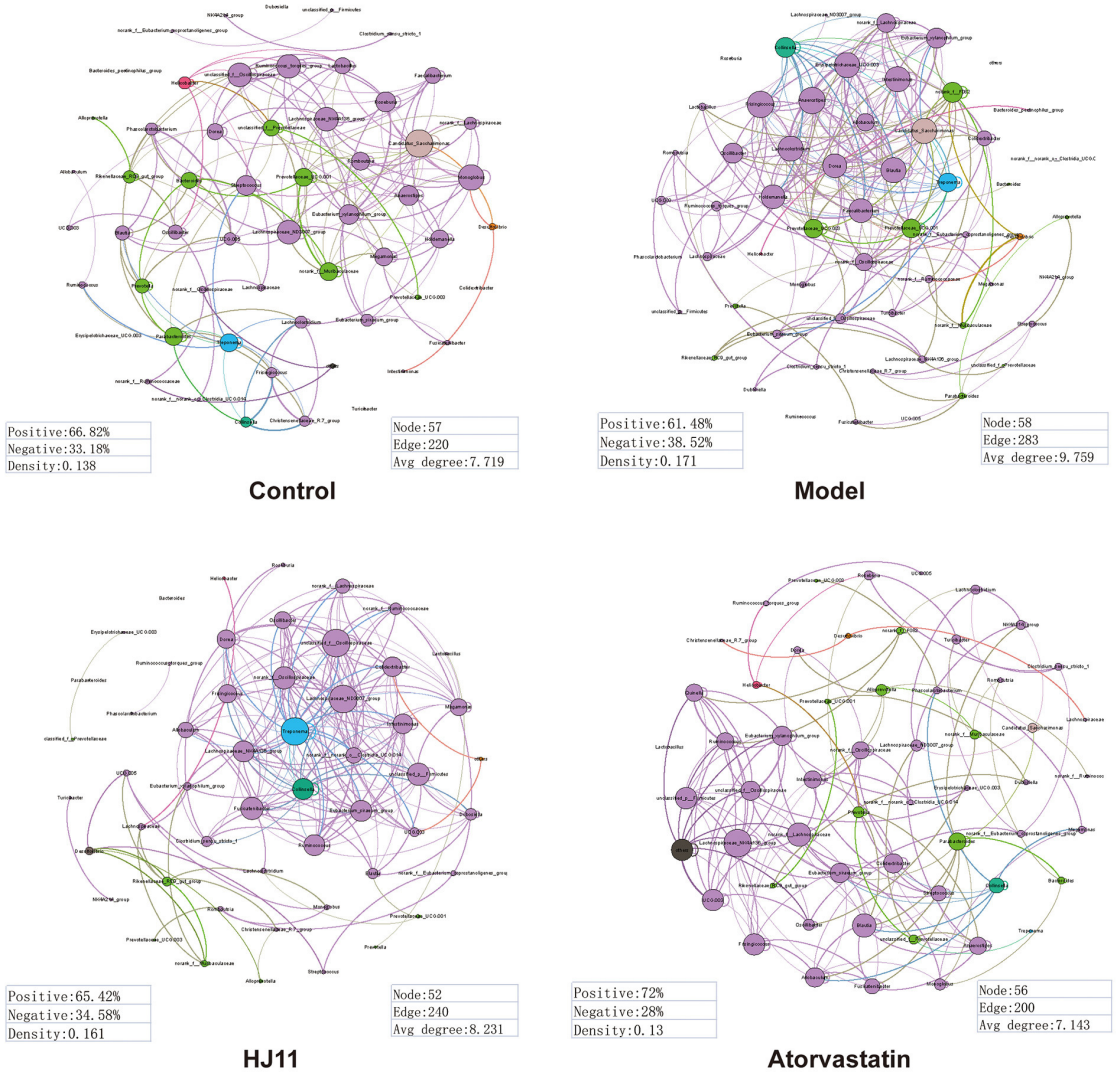
Dominant phylotypes (relative abundance > 0.05) in different groups. Connection indicates strong (Spearman's r > 0.8) and significant (*P* < 0.001) correlation. The co-occurrence network is colored according to the phylum or genus. The size of each node is proportional to the relative abundance of each phylotype. The thickness of each connection between two nodes (edge) is proportional to the Spearman's correlation coefficient. Purple edge indicates positive correlation between two nodes. Green edge indicates negative correlation between two nodes.

We used R and Gephi in a network analysis to unveil the differences in GM complexity among and within groups. The edges and avg degrees corresponded to GM complexity. Figure 5 shows that ACS increased the relative GM complexity, whereas HJ11 decreased it. Thus, HJ11 might affect the GM and play a role in treating ACS.

**Figure 6:**
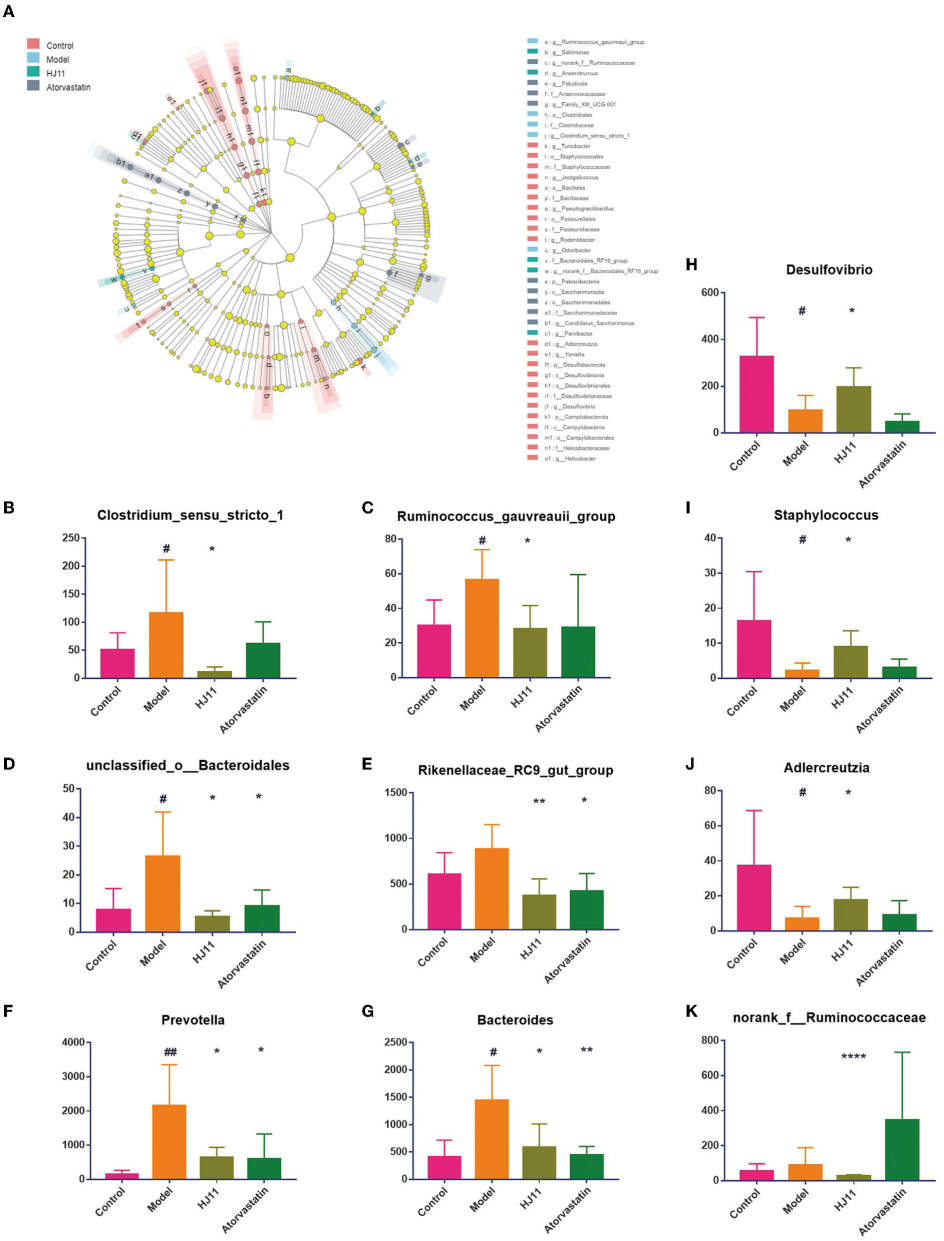
Differentially abundant bacterial taxa. **(A)** Linear discriminant analysis effect size from the phylum to the genus level (LDA score > 2.0). **(B–K)** Relative abundances of gut bacterial genera that were significantly reversed by HJ11 treatment. Student's *t*-test, two-tailed. ^#^*P* < 0.05, ^##^*P* < 0.01 compared with the control group; ^*^P < 0.05, ^**^P < 0.01, ^****^P < 0.0001 compared with the model group.

Linear discriminant analysis effect size (LEfSe) was used to identify the dominant microbial taxa in each group and determine those contributing to the effect of HJ11 on ACS (Figure 6A), forty-one different taxa from the four groups are displayed. The bacterial genera that were inhibited by HJ11 included *norank_f__Ruminococcaceae, Desulfovibrio, Clostridium_sensu_stricto_1, Adlercreutzia, Staphylococcus, Bacteroides, Prevotella, Rikenellaceae_RC9_gut_group, unclassified_o__Bacteroidales*, and *Ruminococcus_gauvreauii_group* (Figures 6B–K).

### 3.6 Metabolite analyses

**Figure 7:**
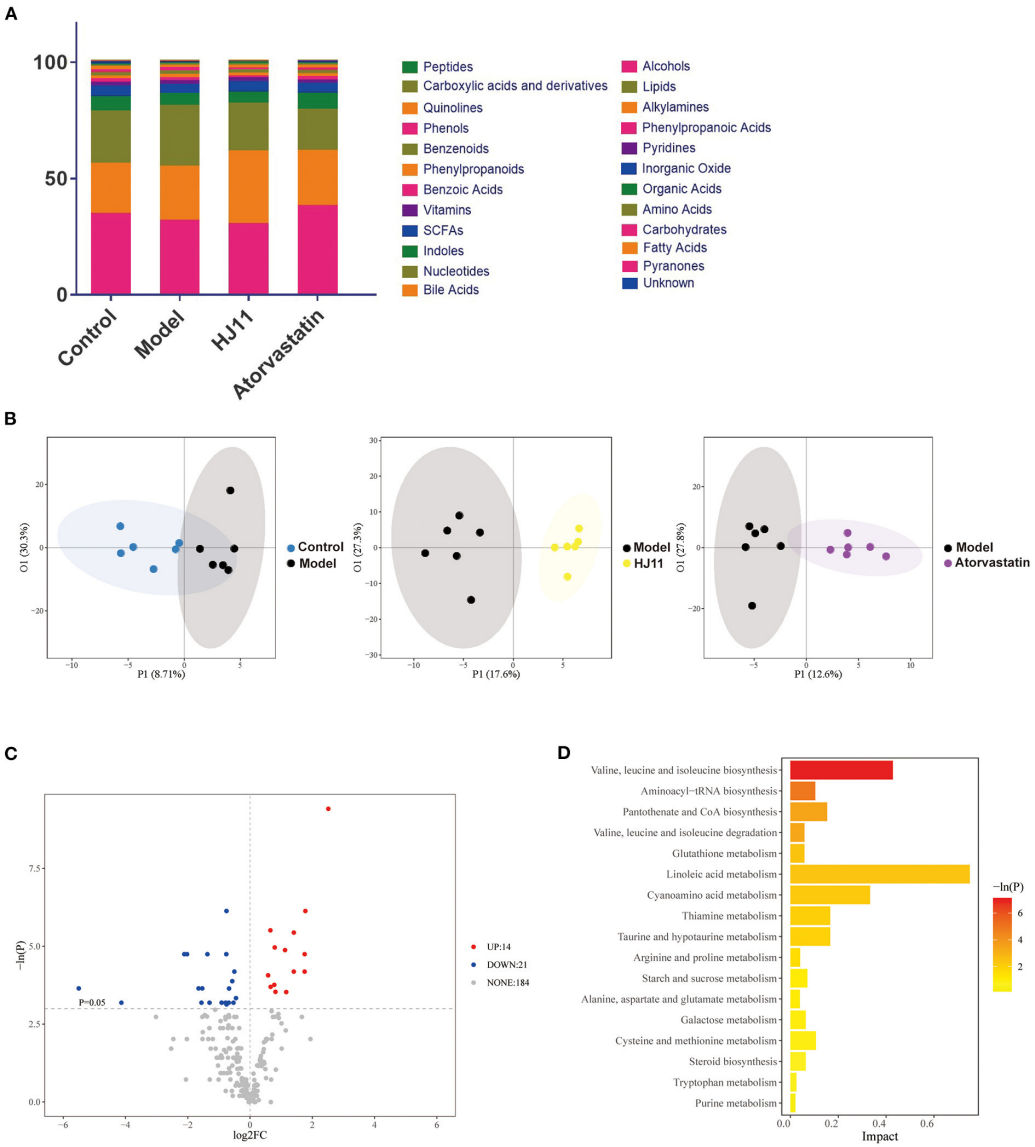
Effects of HJ11 on metabolites in ACS model rats. **(A)** Distribution of metabolites in different groups. **(B)** Orthogonal PLS-DA of metabolomics results in different groups. **(C)** Volcano plot of metabolites in he model and HJ11 groups (screening conditions: log2FC ≥0 and *P* < 0.05). **(D)** Pathway enrichment based on altered metabolites.

A metabolite assay identified 219 metabolites in the intestinal contents. They included carbohydrates, fatty acids, amino acids, organic acids, inorganic oxides, pyridines, lipids, alkylamines, phenylpropanoic acids, and bile acids. The intestinal metabolites varied among groups (Figure 7A). An orthogonal PLS-DA model effectively reduces complexity and was used to screen differentially expressed metabolites (DEMs). The data “points” of each group showed wide separation and spatial distribution, which suggests metabolic differences between groups (Figure 7B).

We used unidimensional statistics (Student's *t*-test) to screen DEMs between the model and HJ11 groups (Figure 7C). There were 14 upregulated and 21 downregulated metabolites. The significant DEMs are listed in Table 2. Based on the relative abundances of the altered metabolites, a KEGG enrichment analysis was conducted and showed that the metabolic pathways of valine, leucine, and isoleucine biosynthesis; aminoacyl-tRNA biosynthesis; pantothenate and CoA biosynthesis; and valine, leucine, and isoleucine degradation were significantly enriched after HJ11 treatment (Figure 7D; with adjusted *P* < 0.05 and ≥2 annotated metabolites). The main enriched pathways were associated with amino acid metabolism. In this pathway,Asymmetric dimethylarginine (ADMA) was the most noticeable metabolism which attracted us. ADMA is an endogenous nitric oxide synthase inhibitor and is associated with endothelial dysfunction, it plays a key role in CVD pathogenesis and atherosclerosis progression (25). Furthermore, ADMA upregulation is an important and independent risk factor for various CVDs (26).

**Table 2:** Differentially expressed metabolites between the model and HJ11 groups.

		**Group**		

**Class**	**Metabolite**	**Model**	**HJ11**	***P***
Alcohols	Octadecanol	2.23 ± 0.80	6.64 ± 2.86	0.0043
	1-Hexadecanol	2.20 ± 0.64	4.79 ± 1.56	0.0076
	Erythritol	3.32 ± 2.72	1.82 ± 1.01	0.0411
Alkylamines	Spermidine	12.67 ± 6.94	2.96 ± 1.14	0.0086
	5-Methoxytryptamine	2.23 ± 1.32	0.84 ± 0.47	0.0259
	3-Amino-2-piperidone	14.61 ± 5.01	8.42 ± 0.54	0.0411
Amino Acids	Lysine	206.87 ± 37.19	121.68 ± 11.87	0.0022
	Isoleucine	107.09 ± 56.26	57.15 ± 12.59	0.0086
	3-Cyanoalanine	1.16 ± 0.65	0.37 ± 0.17	0.0086
	Alanine	252.58 ± 95.64	162.42 ± 18.96	0.0151
	Asymmetric dimethylarginine	25.34 ± 6.20	17.07 ± 2.36	0.0206
	Cysteine	5.59 ± 1.59	3.50± 1.04	0.0261
	Valine	177.24 ± 73.04	106.62 ± 33.74	0.0411
	4-Hydroxyproline	4.68 ± 1.69	2.78 ± 0.97	0.0434
Bile acids	Lithocholic acid	12.93 ± 5.98	22.21 ± 6.03	0.0232
Carbohydrates	Glucaric acid	0.63 ± 0.23	1.09 ± 0.24	0.0069
	1,5-Anhydroglucitol	15.43 ± 6.96	24.43 ± 3.81	0.0247
	Gluconic acid	1.49 ± 2.92	0.14 ± 0.09	0.0411
	Sucrose	2.90 ± 1.91	0.99 ± 1.30	0.0411
	Turanose	4.66 ± 2.68	2.40 ± 3.61	0.0411
Carboxylic acids and derivatives	gly_pro	4.71 ± 1.95	2.51 ± 1.02	0.0411
Fatty acids	Arachidic acid	44.43 ± 22.09	129.35 ± 89.41	0.0151
	Nonadecanoic acid	3.23 ± 0.99	5.7 ± 2.00	0.0290
	Heptadecanoic acid	23.48 ± 11.51	52.6 ± 23.66	0.0292
	Linoleic acid	136.38 ± 23.69	99.86 ± 28.00	0.0355
Indoles	Indolelacetic acid	2.58 ± 2.09	0.69 ± 1.02	0.0259
	5-Hydroxyindoleacetic acid	9.14 ± 6.20	2.73 ± 1.98	0.0259
Lipids	Lathosterol	8.62 ± 1.76	4.45 ± 2.09	0.0086
Nucleotides	Pseudouridine	1.59 ± 0.94	4.29 ± 1.82	0.0086
	Xanthine	3.17 ± 1.45	11.82 ± 8.09	0.0151
Organic acids	Methylphosphate	9.33 ± 6.12	53.34 ± 12.32	8.13E-05
	3-Methyl-2-oxopentanoic acid	2.48 ± 1.03	8.39 ± 3.09	0.0022
	3,4-Dihydroxybutyric acid	1.57 ± 0.79	0.91 ± 0.43	0.0411
Phenylpropanoids	Caffeic acid	0.88 ± 0.25	1.39 ± 0.21	0.0040
Vitamins	δ-Tocopherol	0.44 ± 0.09	0.67 ± 0.16	0.0171

### 3.7 Correlations among immunity indices, cardiac function, intestinal metabolites, and GM

**Figure 8:**
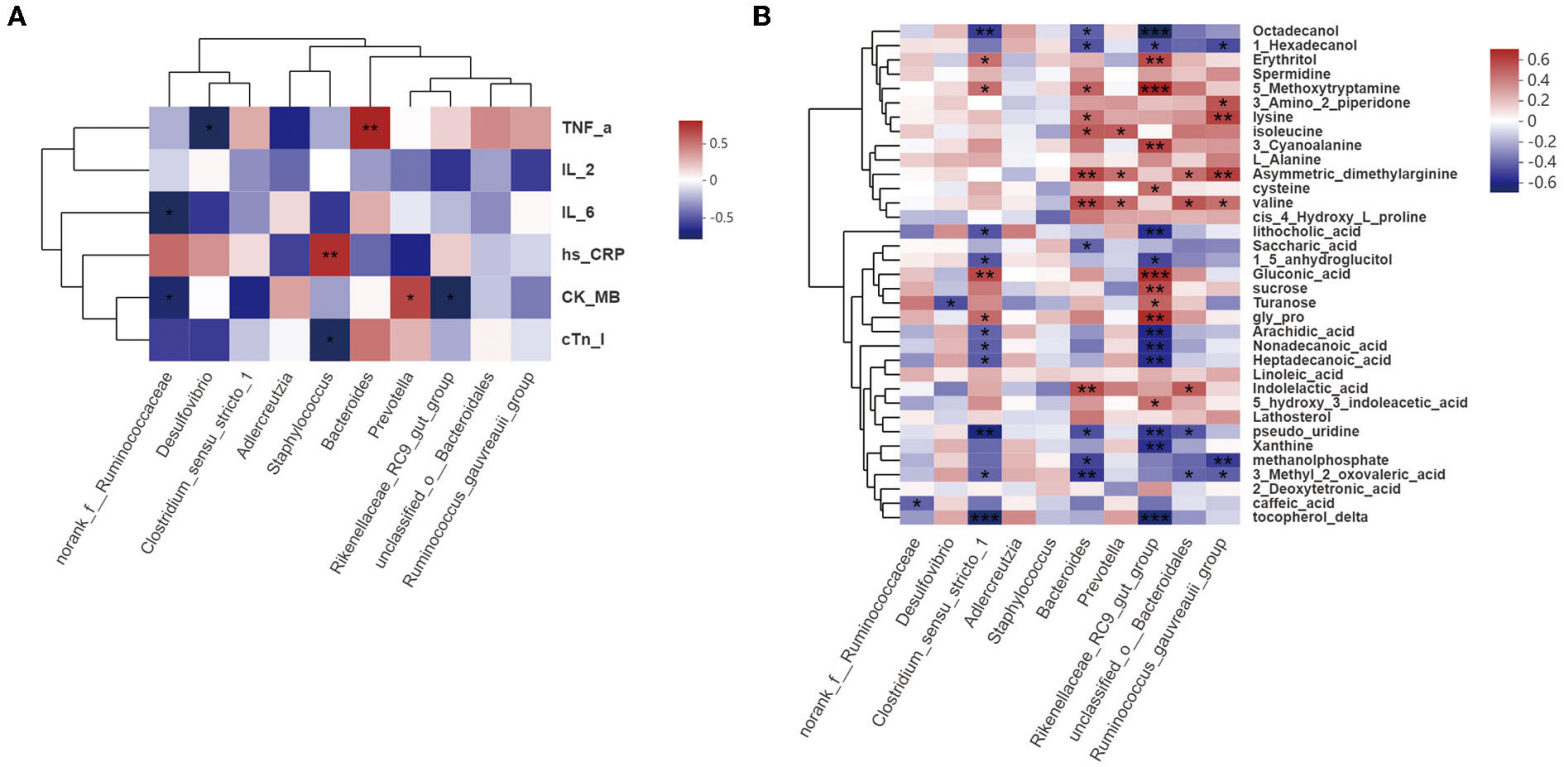
Correlation analysis. **(A)** Correlations among gut bacteria, proinflammatory factors (TNF-α, IL-2, IL-6, and hs-CRP), and cardiac function indices (CK-MB and cTnI) (Spearman's r >0.1 or <0.1; *n* = 6/group). **(B)** Correlations among gut bacteria and metabolites (Spearman's r >0.1 or <0.1; *n* = 6/group). ^*^P < 0.05, ^**^P < 0.01, ^***^P < 0.001.

Correlations among gut bacteria, inflammatory indices, cardiac function indices, and intestinal metabolites were determined *via* Spearman's rank correlation to identify ACS-associated bacterial taxa and metabolites affected by HJ11 treatment. Figure 8A shows that TNF-α levels were significantly correlated with increasing *Bacteroides* and decreasing *Desulfovibrio* abundance. The abundance of the genus *norank_f__Ruminococcaceae* was negatively correlated with IL-6 levels. *Staphylococcus* abundance was positively correlated with hs-CRP levels and negatively correlated with cTnI levels. Levels of the cardiac function indicator CK-MB were positively correlated with the abundance of *Prevotella* and negatively correlated with that of *norank_f__Ruminococcaceae* and *Rikenellaceae_RC9_gut_group*.

Figure 8B shows that *norank_f__Ruminococcaceae, Desulfovibrio, Clostridium_sensu_stricto_1, Bacteroides, Prevotella, Rikenellaceae_RC9_gut_group, unclassified_o__ Bacteroidales*, and *Ruminococcus_gauvreauii_group* abundances were significantly correlated with the metabolite profile. *Bacteroides* and *Rikenellaceae_RC9_gut_group* abundances were associated with the levels of most metabolites, and with CVD and its risk factors, such as hypertension and hyperlipidemia. ADMA levels were also significantly correlated with *Bacteroides* abundance.

These findings suggest that, overall, the increased ACS-ameliorating effect of HJ11 may be associated with specific bacterial taxa and metabolic pathway alterations, with *Bacteroides* and *Rikenellaceae_RC9_gut_group* potentially playing important roles in this process. HJ11 may increase the abundance of beneficial bacteria related to CVDs and their risk factors, alter metabolite profiles, lower ADMA levels, and be efficacious in the treatment of ACS.

## 4. Discussion

Inflammation is a key factor in ACS development. The TCM theory proposes that inflammation is closely related to the pathological changes in heat and toxicity, and treatment is based on the principle of “clearing camp and cooling blood, detoxifying and dispersing nodules” (27). In this study, we ligated the left anterior descending branch of the coronary artery to trigger ST-segment elevation and establish the ACS model (24), and examined the therapeutic efficacy of HJ11 using atorvastatin as a positive control. In model rats, atorvastatin therapy significantly lowers plasma CK-MB and lactate dehydrogenase levels (28). Moreover, pretreatment with atorvastatin significantly reduces plasma IL-6 and TNF-α levels as well as Rho-kinase activity, myocardial infarct size, and cardiomyocyte apoptosis (29). Studies have shown that the intestinal microflora is altered during the development and treatment of various diseases, and participates in numerous physiological processes, including the inflammatory response, and energy and nutrient metabolism (30). Here, we examined inflammation-related indices in ACS model rats and found that HJ11 exerted anti-inflammatory effects and improved cardiac function. We also integrated microbiomics and metabolomics analyses to reveal alterations in the intestinal microflora and metabolites induced by HJ11. We discovered that HJ11 therapy partially normalized the ACS-induced intestinal microflora and metabolite profile disruption.

The components of TCM therapeutics are complex, and their quality widely varies with the plant source, production area, local climate, among others. To ensure the quality of Chinese herbs, we used the Chinese Pharmacopeia 2020 standards and HPLC to determine their content and purity of active ingredients. All herb materials used in this study met the requirements.

Obesity is a risk factor for heart disease. Every 1 kg/m^2^ gain increases the risk of heart failure by 5–7%. In addition, the risk of heart failure is twice as high in patients with obesity compared to that in those with normal body mass index (31). Here, HJ11 significantly reduced the weight gain in ACS model rats, suggesting that it might lower the risk of heart disease by slowing down weight gain.

We used a small animal ultrasound imaging system (32) to measure EF and FS. Left ventricular internal end-diastolic dimension and -systolic dimension affected EF and FS. We also found that HJ11 improved cardiac function in ACS rats, which was consistent with previously reported findings (33–35). Moreover, HE staining showed that HJ11 ameliorated or mitigated disorganized myocardial tissue, broken myocardial bundles, and myocardial cell degeneration and damage. Taken together, these results indicate that HJ11 consistently improved heart function.

Proinflammatory factors can play a role in pathogenesis and promote disease development, contributing to the “inflammatory load” (36, 37). IL-2, IL-6, TNF-α, and hs-CRP promote inflammation, thrombosis, matrix degradation, and apoptosis and are implicated in ACS progression (38–41). hs-CRP has been found to be positively correlated with ACS and its prognosis (38). Herein, HJ11 lowered the serum IL-2, TNF-α, and hs-CRP levels and had a pronounced effect on inflammation. Myocardial CK-MB and cTnI are specific injury-sensitive indices of severe myocardial tissue damage. They are released into the bloodstream, and their serum levels are important diagnostic and prognostic criteria for ACS (42, 43). HJ11 also reduced CK-MB and cTnI levels in our study. Furthermore, echocardiography and HE staining of cardiac tissues showed the cardioprotective effect of HJ11. The foregoing results suggest that HJ11 administration for ACS treatment is efficacious.

The intestinal microflora affects the inflammatory state of blood vessels. Vascular inflammation may induce atherosclerosis, promote insulin resistance, and elevate blood pressure through several metabolic and inflammatory pathways (42, 43). Thus, restoring the intestinal microflora homeostasis may help combat CVD. Prior research on the intestinal microflora associated with CVD showed that the F/B ratio is altered under this condition relative to normal controls (44, 45). Here, we found that the intestinal microflora abundance and diversity were significantly reduced in the ACS model group, and that HJ11 treatment improved the F/B ratio.

We also found that HJ11 had a restorative effect on the intestinal microflora at the genus level. *Norank_f__Ruminococcaceae, Bacteroides, Rikenellaceae_RC9_gut_group, Prevotella, Staphylococcus*, and *Desulfovibrio* abundances were significantly correlated with the serum TNF-α, IL-6, hs-CRP, CK-MB, and cTnI levels. *Rikenellaceae_RC9_gut_group* predominates in the Bacteroidetes phylum. However, only few studies have associated *Rikenellaceae_RC9_gut_group* with CVD, hyperlipidemia, and other heart diseases and related factors. Significant alterations in *Rikenellaceae_RC9_gut_group* may contribute to the pathogenesis of acute myocardial ischemia by affecting intestinal permeability, oxidative stress, and energy metabolism (46). Moreover, *Rikenellaceae_RC9_gut_group* was positively correlated with high fat diet-induced “harmful indicators” and negatively correlated with “beneficial indicators” (47). The abundance of *Rikenellaceae_RC9_gut_group* was also found to influence the interaction between vitamin A and Toll-like receptor 4 (48). High fat diet is a risk factor for ACS, it significantly increases the abundance of *unclassified_o_Bacteroidales*, whereas drug therapy decreases it (47). The cardiovascular outcomes of patients with ST-segment elevation myocardial infarction are determined by the translocation of intestinal microbiota into systemic circulation. *Bacteroides* is significantly more abundant in patients with ST-segment elevation myocardial infarction than in normal controls. Patients with ST-segment elevation myocardial infarction may also present tight junction disruptions in their gut barriers (49). *Prevotella* abundance is relatively higher in pre-hypertensive and hypertensive patients than in healthy controls (50). *Prevotella* might also contribute to atherosclerosis progression (51). *Staphylococcus* has been detected in lesions and the ilea of patients with atherosclerosis. Hence, GM is likely to participate in atherosclerosis development (52).

Gut microorganisms can produce a wide variety of metabolites that regulate different physiological processes. GM-produced metabolites have both positive and negative effects on the body. Metabolomics has been widely used to elucidate CVD pathogenesis (53, 54). Here, we identified 23 DEMs and four experimentally relevant metabolic pathways. Bile acids are cholesterol metabolites formed in the liver and play important roles in lipid metabolism (55). The latter responds to cholesterol metabolism and is associated with *Bifidobacterium* and *Lactobacillus* abundance (56). We found that bile acid levels were lower in the ACS model group and significantly higher in the HJ11 group than in the control group (*P* < 0.05). Intestinal *Escherichia coli* breaks down amino acids and produces indole-like substances that can induce left ventricular hypertrophy and increase the risk of CVD (57). We found that 5-hydroxyindoleacetic acid, indolelactic acid, and serotonin levels were significantly (*P* < 0.05) elevated in the ACS model group and reduced in the HJ11 group relative to the control group. *Bacteroides* produces various metabolites, such as ADMA and indoleacetic acid, that are associated with CVD (58). ADMA attenuates nitric oxide, enhances the production of reactive oxidants, and is an endogenous nitric oxide synthase inhibitor (59, 60), thus playing key roles in CVD progression. A high serum ADMA level is an important independent risk factor for various CVDs, and can be accounted for the observed increases in cardiovascular morbidity and mortality in patients with chronic kidney disease, rheumatoid arthritis, vasculitis, among others (61, 62). Several drugs (such as statins) that are administered for the management of CVD and type 2 diabetes mellitus lower serum ADMA levels in tissue fibrosis pathogenesis (60). Here, we found that HJ11 reduced ADMA levels in the ACS model. This mechanism as well as intestinal microflora modulation partially explain the observed effects of HJ11 on ACS. But could HJ11 regulate the GM and metabolites first then give play to treat ACS, still need further study. Moreover, the direct effect of ADMA on the endothelial dysfunction also should be clarified. Also when HJ11 is used on ACS in clinical treatment, clinical trails are needed to determine the specific dose, the time, etc., so as to better treating ACS.

This study showed that HJ11 exerts anti-inflammatory effects and improves cardiac function in ACS. Microbiome and metabolomics analyses demonstrated that HJ11 affected the GM structure and their metabolites. Overall, HJ11 seems to increase the relative abundance of beneficial bacterial genera such as *Bacteroides* and *Rikenellaceae_RC9_gut_group*, mitigate the risk factors associated with CVD, alter microbial metabolites, lower ADMA levels, and effectively treat ACS. Nevertheless, the detailed modes of action of HJ11 in ACS therapy remain to be clarified.

## Data availability statement

The data presented in the study are deposited in the NCBI repository (https://www.ncbi.nlm.nih.gov/), accession number PRJNA912125.

## Ethics statement

The animal study was reviewed and approved by Animal Ethics Committee of the Institute of Basic Theory for Chinese Medicine CACMS.

## Author contributions

DB and JH contributed to the study conception and design. NZ analyzed data and wrote the manuscript. YW acquired data. YM revised the manuscript. XZ performed HPLC. XL, YG, YD, NZ, and YW conducted the animal experiments. All authors agreed to the journal to which the article has been submitted and to be accountable for all aspects of the work and made significant contributions to this work.

## Footnotes

^1^ https://github.com/OpenGene/fastp

^2^ http://www.cbcb.umd.edu/software/flash

^3^ http://drive5.com/uparse/

^4^ http://rdp.cme.msu.edu/

^5^ https://github.com/picrust/picrust2
